# Data on the association between age at natural menopause and physical function in older women from the International Mobility in Aging Study (IMIAS)

**DOI:** 10.1016/j.dib.2019.103811

**Published:** 2019-03-06

**Authors:** M.P. Velez, N. Rosendaal, B. Alvarado, S. da Câmara, E. Belanger, C.M. Pirkle

**Affiliations:** a Division of Reproductive Endocrinology and Infertility, Department of Obstetrics and Gynecology, Queen's University, Victory 4, 76 Stuart St. Kingston, ON, K7L 2V7, Canada; b Department of Public Health Sciences, Queen's University, Carruthers Hall 2nd Floor, 62 Fifth Field Company Lane, Kingston, ON, K7L 3N6, Canada; c Faculty of Health Sciences of Trairí, Universidade Federal do Rio Grande do Norte, Rua Teodorico Bezerra, 2-122, Santa Cruz-RN, 59200-000, Brazil; d Center for Gerontology and Healthcare Research, Department of Health Services, Policy and Practice, School of Public Health, Brown University, 121 South Main Street, Providence, RI, 02903, USA; e Office of Public Health Studies, University of Hawai'i at Mānoa, 1960 E West Rd, Biomedical Bldg D104H, Honolulu, HI, 96822, USA

## Abstract

Women experience worse physical function and greater physical decline than men at similar ages. These sex differences are heterogeneous across settings and plausibly linked to gender inequality, with evidence of increasing disadvantage for women in increasingly iniquitous societies. As described in “Age at natural menopause and physical function in older women from Albania, Brazil, Colombia and Canada: A life-course perspective” [Velez et al., 2019] we assessed the association between age at natural menopause (ANM) and objectives markers of physical function (i.e., gait speed and grip strength) in older women from the International Mobility in Aging Study (IMIAS). For all sites combined, women with ANM ≥55 had higher gait speed than those with ANM 50–54. Women with ANM <40 had significantly lower grip strength compared with all other groups. In this article, we describe the region-specific associations between ANM, gait speed, and grip strength in 775 women aged 65–74, from the Southeastern European site (Tirana, Albania), Latin American sites (Manizales, Colombia and Natal, Brazil), and Canadian sites (Kingston, Ontario and Saint-Hyacinthe, Quebec). In region-specific analyses, ANM was associated with grip strength in Albania and Latin America and with gait speed in Albania only. No associations were observed in Canada.

**Table:** Specifications table

Subject area	*Obstetrics, Gynecology and Women's Health*
More specific subject area	*Menopause*
Type of data	*Tables*
How data was acquired	*Population-based prospective cohort study*
Data format	*Analyzed*
Experimental factors	Data came from the sub-sample of women from the International Mobility in Aging Study (IMIAS), categorized by research site.
Experimental features	Association between age at natural menopause and measures of physical function (gait speed, grip strength), taking into account socioeconomic factors (education, income, and socio-economic and social adversities and childhood, smoking, BMI, hormone replacement therapy)
Data source location	Tirana (Albania), Natal (Brazil), Manizales (Colombia), Kingston (Ontario, Canada), and Saint-Hyacinthe (Quebec, Canada)
Data accessibility	*Data are with this article*
Related research article	Velez M.P, Rosendaal N, Alvarado B, da Câmara S, Belanger E, Pirkle C. Age at natural menopause and physical function in older women from Albania, Brazil, Colombia and Canada: A life-course perspective. Maturitas 2019 April; 122: 22–30. doi.org/10.1016/j.maturitas.2018.12.015[1]

**Table:** 

**Value of the data** • The data can be used to understand the association between ANM and measures of physical function in an international, community-based sample of older women from five diverse contexts (Albania, Brazil, Colombia and Canada). • These results may also be useful in comparative work with other cohorts to better understand how life-course events modify the association between ANM and physical function in diverse contexts. • Our model of analyses can be used for other researchers interested in the assessment of the association between ANM and physical function from a life-course perspective in diverse contexts.

## 1 Data

The data presented in this article are tables of analyzed data form the International Mobility in Aging Study (IMIAS), a population-based prospective cohort study amongst community-dwelling older adults with sites in 5 cities: 2 in Canada (Kingston and Saint-Hyacinthe), and one in each of the following: Colombia (Manizales), Brazil (Natal), and Albania (Tirana). Funded by the Canadian Institutes of Human Research (CIHR), as part of the Institute of Aging strategic initiative on mobility in aging, IMIAS includes substantial socio-economic and reproductive history data (e.g., age at natural menopause, pregnancy history, use of hormone replacement therapy). In addition, IMIAS collected objective measures of physical function (i.e., gait speed and grip strength) in women aged 65–74. A detailed description of the recruitment and study procedures for the IMIAS can be obtained from Gomez et al. [2] Institutional ethics review board approval was obtained from the participating sites. Written informed consent was obtained from all participants. In this paper we present the following region-specific results: Population characteristics by ANM (Tables 1A, 1B, 1C); distribution (or mean) of ANM and population characteristics by gait speed (Table 2A); and grip strength (Table 2B). We also present multivariate linear regression models of the association between ANM and gait speed (Table 3A), and ANM and grip strength (Table 4A).

## 2 Experimental design, materials, and methods

### 2.1 Study population

The IMIAS study has been described elsewhere [2]. The study website contains details about the study design, data collection, sample strategies, researchers, publications, and contact information for researchers interested in conducting analyses with the IMIAS data. http://www.imias.ufrn.br/about.htm.

### 2.2 Methods and statistical analysis

The exposure variable, Age at Natural Menopause was obtained by questionnaire data, and recorded in years. The outcomes, Gait speed and grip strength, were obtained using standardized protocols as described in detail in the Short Physical Performance Battery (SPPB) website (http://www.grc.nia.nih.gov/branches/leps/sppb/index.htm).

Details about covariates, and categories used according to each region are described in Ref. [1].

Descriptive analyses included site-specific comparison of covariates by age at menopause, and gait speed and grip strength using chi2 for categorical variables, and one-way ANOVA for continuous variables. Linear regressions were performed to assess the relationships between ANM and gait speed and grip strength specific to each region. To select the covariates relevant to each region and obtain the most parsimonious model, we used a backwards selection procedure. Statistical analysis was conducted using STATA, version 14..

## Financial support

This work was supported by CIHR (Canadian Institute for Health Research) grant: ACD 151286.

## Author contributions

Author contributions for this project are detailed in Ref. [1].
