# Supplementary material for: Data on the association between age at natural menopause and physical function in older women from the International Mobility in Aging Study (IMIAS)
Source: Data Brief. 2019 Mar 6;23:103811. doi: 10.1016/j.dib.2019.103811 (PMC6660581; doi:10.1016/j.dib.2019.103811)
Supplement: Multimedia component 1 [file mmc1.docx]

**Data from the International Mobility in Aging Study (IMIAS) relating Age at natural menopause and physical function in older women from diverse research sites**

Velez M.P , Rosendaal N, Alvarado B, da Câmara S, Belanger E, Pirkle C

We wish to confirm that there are no known conflicts of interest associated with this publication and there has been no significant financial support for this work that could have influenced its outcome.

We confirm that the manuscript has been read and approved by all named authors and that there are no other persons who satisfied the criteria for authorship but are not listed. We further confirm that the order of authors listed in the manuscript has been approved by all of us.

We confirm that we have followed the regulations of our institutions concerning intellectual property.

We further confirm that any aspect of the work covered in this manuscript that has involved human patients has been conducted with the ethical approval of all relevant bodies and that such approvals are acknowledged within the manuscript.

We understand that the Corresponding Author is the sole contact for the Editorial process (including Editorial Manager and direct communications with the office). She is responsible for communicating with the other authors about progress, submissions of revisions and final approval of proofs.


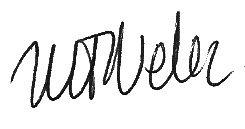
Signed by Dr. Maria Velez on behalf of co-authors
