# Supplementary material for: Data on the association between age at natural menopause and physical function in older women from the International Mobility in Aging Study (IMIAS)
Source: Data Brief. 2019 Mar 6;23:103811. doi: 10.1016/j.dib.2019.103811 (PMC6660581; doi:10.1016/j.dib.2019.103811)
Supplement: Multimedia component 2 [file mmc2.docx]

# Supplemental material:

Table 1A: Population characteristics by age at natural menopause, region-specific – Canada

| Characteristics | | Age at natural Age at natural menopause - categories menopause - categories | | | | | | | | | | P-value |
| --- | --- | --- | --- | --- | --- | --- | --- | --- | --- | --- | --- | --- |
|  |  | <40 (N=9) | | 40-44 (N=23) | | 45-49 (N=70) | | 50-54 (N=118) | | ≥55 (N=67) | |  |
| **Study site, N, %** | |  |  |  |  |  |  |  |  |  |  |  |
|  | Kingston | 6 | *3.8%* | 14 | *8.8%* | 35 | *21.9%* | 67 | *41.9%* | 38 | *23.8%* | 0.792 |
|  | St Hyacinthe | 3 | *2.4%* | 9 | *7.1%* | 35 | *27.6%* | 51 | *40.2%* | 29 | *22.8%* |  |
| **Age, mean, SD** | | 70.00 | *2.92* | 68.35 | *2.44* | 68.86 | *2.55* | 68.56 | *2.58* | 69.24 | *2.56* | 0.227 |
| **Education, site specific tertiles, N, %** | |  |  |  |  |  |  |  |  |  |  |  |
|  | lowest tertile | 5 | *4.2%* | 16 | *13.4%* | 29 | *24.4%* | 43 | *36.1%* | 26 | *21.8%* | 0.092 |
|  | middle tertile | 3 | *2.9%* | 4 | *3.9%* | 29 | *28.4%* | 40 | *39.2%* | 26 | *25.5%* |  |
|  | highest tertile | 1 | *1.5%* | 3 | *4.5%* | 12 | *18.2%* | 35 | *53.0%* | 15 | *22.7%* |  |
| **Income, N, %** | |  |  |  |  |  |  |  |  |  |  |  |
|  | poor | 5 | *5.3%* | 11 | *11.6%* | 29 | *30.5%* | 33 | *34.7%* | 17 | *17.9%* | 0.095 |
|  | middle | 1 | *1.0%* | 8 | *7.7%* | 20 | *19.2%* | 45 | *43.3%* | 30 | *28.8%* |  |
|  | middle high/high | 3 | *4.2%* | 3 | *4.2%* | 15 | *21.1%* | 35 | *49.3%* | 15 | *21.1%* |  |
| **Height, mean, SD** | | 160.22 | *2.54* | 159.96 | *5.75* | 158.46 | *6.32* | 159.77 | *6.43* | 160.1 | *5.20* | 0.506 |
| **BMI, N, %** | |  |  |  |  |  |  |  |  |  |  |  |
|  | Normal or underweight (<25) | 3 | *3.1%* | 8 | *8.2%* | 28 | *28.9%* | 39 | *40.2%* | 19 | *19.6%* | 0.718 |
|  | Overweight (25.0-29.9) | 2 | *1.8%* | 10 | *8.8%* | 22 | *19.5%* | 49 | *43.4%* | 30 | *26.5%* |  |
|  | Obese (>=30) | 4 | *5.2%* | 5 | *6.5%* | 20 | *26.0%* | 30 | *39.0%* | 18 | *23.4%* |  |
| **Hysterectomy (post-menopausal), N, %** | |  |  |  |  |  |  |  |  |  |  |  |
|  | Had hysterectomy | 4 | *11.4%* | 3 | *8.6%* | 15 | *42.9%* | 9 | *25.7%* | 4 | *11.4%* | 0.001 |
|  | Did not have hysterectomy | 5 | *2.0%* | 20 | *7.9%* | 55 | *21.8%* | 109 | *43.3%* | 63 | *25.0%* |  |
| **HRT, ever, N, %** | |  |  |  |  |  |  |  |  |  |  |  |
|  | no | 6 | *5.3%* | 10 | *8.8%* | 29 | *25.7%* | 42 | *37.2%* | 26 | *23.0%* | 0.432 |
|  | yes | 3 | *1.7%* | 13 | *7.5%* | 41 | *23.6%* | 76 | *43.7%* | 41 | *23.6%* |  |
| **Smoking, last 15 years N, %** | |  |  |  |  |  |  |  |  |  |  |  |
|  | no | 8 | *3.0%* | 19 | *7.1%* | 64 | *23.8%* | 112 | *41.6%* | 66 | *24.5%* | 0.069 |
|  | yes | 1 | *5.6%* | 4 | *22.2%* | 6 | *33.3%* | 6 | *33.3%* | 1 | *5.6%* |  |
| **Childhood economic adversity, N, %** | |  |  |  |  |  |  |  |  |  |  |  |
|  | No adversities | 5 | *2.5%* | 17 | *8.5%* | 47 | *23.5%* | 85 | *42.5%* | 46 | *23.0%* | 0.809 |
|  | One or more adversities | 4 | *4.6%* | 6 | *6.9%* | 23 | *26.4%* | 33 | *37.9%* | 21 | *24.1%* |  |
| **Childhood social adversity, N, %** | |  |  |  |  |  |  |  |  |  |  |  |
|  | No adversities | 4 | *2.0%* | 14 | *6.9%* | 48 | *23.8%* | 87 | *43.1%* | 49 | *24.3%* | 0.299 |
|  | One or more adversities | 5 | *5.9%* | 9 | *10.6%* | 22 | *25.9%* | 31 | *36.5%* | 18 | *21.2%* |  |
| **Teenage pregnancy, N, %** | |  |  |  |  |  |  |  |  |  |  |  |
|  | No teenage pregnancy | 9 | *3.4%* | 19 | *7.2%* | 63 | *23.9%* | 112 | *42.4%* | 61 | *23.1%* | 0.253 |
|  | Teenage pregnancy (<20) | 0 | *0.0%* | 4 | *17.4%* | 7 | *30.4%* | 6 | *26.1%* | 6 | *26.1%* |  |
| **Parity, N, %** | |  |  |  |  |  |  |  |  |  |  |  |
|  | Nulliparous | 0 | *0.0%* | 2 | *4.3%* | 17 | *36.2%* | 19 | *40.4%* | 9 | *19.1%* | 0.206 |
|  | 1 to 3 births | 9 | *4.5%* | 16 | *8.0%* | 46 | *22.9%* | 85 | *42.3%* | 45 | *22.4%* |  |
|  | 4 or more births | 0 | *0.0%* | 4 | *10.5%* | 7 | *18.4%* | 14 | *36.8%* | 13 | *34.2%* |  |

* 17 missing values in income, 1 missing value in parity

P-values: chi2 for categorical/dichotomous covariates, anova for continuous covariates.

Table 1B: Population characteristics by age at natural menopause, region-specific – Latin America

| Characteristics | | Age at natural menopause - categories | | | | | | | | | | P-value |
| --- | --- | --- | --- | --- | --- | --- | --- | --- | --- | --- | --- | --- |
|  |  | <40 (N=16) | | 40-44 (N=43) | | 45-49 (N=110) | | 50-54 (N=102) | | ≥55 (N=41) | |  |
| **Study site, N, %** | |  |  |  |  |  |  |  |  |  |  |  |
|  | Manizales | 9 | *5.7%* | 20 | *12.7%* | 55 | *35.0%* | 51 | *32.5%* | 22 | *14.0%* | 0.955 |
|  | Natal | 7 | *4.5%* | 23 | *14.8%* | 55 | *35.5%* | 51 | *32.9%* | 19 | *12.3%* |  |
| **Age, mean, SD** | | 69.81 | *3.08* | 69.84 | *3.02* | 69.31 | *2.68* | 68.75 | *2.62* | 68.83 | *2.80* | 0.158 |
| **Education, site specific tertiles, N, %** | |  |  |  |  |  |  |  |  |  |  |  |
|  | lowest tertile | 7 | *5.5%* | 19 | *14.8%* | 37 | *28.9%* | 52 | *40.6%* | 13 | *10.2%* | 0.209 |
|  | middle tertile | 4 | *3.5%* | 17 | *14.9%* | 44 | *38.6%* | 30 | *26.3%* | 19 | *16.7%* |  |
|  | highest tertile | 5 | *7.1%* | 7 | *10.0%* | 29 | *41.4%* | 20 | *28.6%* | 9 | *12.9%* |  |
| **Income, N, %** | |  |  |  |  |  |  |  |  |  |  |  |
|  | poor | 8 | *6.5%* | 13 | *10.6%* | 42 | *34.1%* | 41 | *33.3%* | 19 | *15.4%* | 0.458 |
|  | middle | 6 | *4.1%* | 26 | *17.6%* | 50 | *33.8%* | 51 | *34.5%* | 15 | *10.1%* |  |
|  | middle high/high | 2 | *4.9%* | 4 | *9.8%* | 18 | *43.9%* | 10 | *24.4%* | 7 | *17.1%* |  |
| **Height, mean, SD** | | 150.5 | *5.48* | 150.77 | *5.23* | 150.15 | *5.56* | 151.12 | *5.75* | 151 | *5.10* | 0.765 |
| **BMI, N, %** | |  |  |  |  |  |  |  |  |  |  |  |
|  | Normal or underweight (<25) | 5 | *5.7%* | 12 | *13.6%* | 29 | *33.0%* | 30 | *34.1%* | 12 | *13.6%* | 0.909 |
|  | Overweight (25.0-29.9) | 7 | *5.0%* | 20 | *14.2%* | 53 | *37.6%* | 40 | *28.4%* | 21 | *14.9%* |  |
|  | Obese (>=30) | 4 | *4.8%* | 11 | *13.3%* | 28 | *33.7%* | 32 | *38.6%* | 8 | *9.6%* |  |
| **Hysterectomy (post-menopausal), N, %** | |  |  |  |  |  |  |  |  |  |  |  |
|  | Had hysterectomy | 2 | *5.3%* | 4 | *10.5%* | 14 | *36.8%* | 14 | *36.8%* | 4 | *10.5%* | 0.936 |
|  | Did not have hysterectomy | 14 | *5.1%* | 39 | *14.2%* | 96 | *35.0%* | 88 | *32.1%* | 37 | *13.5%* |  |
| **HRT, ever, N, %** | |  |  |  |  |  |  |  |  |  |  |  |
|  | no | 12 | *4.6%* | 39 | *14.9%* | 89 | *34.1%* | 87 | *33.3%* | 34 | *13.0%* | 0.515 |
|  | yes | 4 | *7.8%* | 4 | *7.8%* | 21 | *41.2%* | 15 | *29.4%* | 7 | *13.7%* |  |
| **Smoking, last 15 years N, %** | |  |  |  |  |  |  |  |  |  |  |  |
|  | no | 15 | *5.3%* | 35 | *12.3%* | 101 | *35.6%* | 93 | *32.7%* | 40 | *14.1%* | 0.123 |
|  | yes | 1 | *3.6%* | 8 | *28.6%* | 9 | *32.1%* | 9 | *32.1%* | 1 | *3.6%* |  |
| **Childhood economic adversity, N, %** | |  |  |  |  |  |  |  |  |  |  |  |
|  | No adversities | 5 | *3.3%* | 21 | *13.8%* | 56 | *36.8%* | 50 | *32.9%* | 20 | *13.2%* | 0.705 |
|  | One or more adversities | 11 | *6.9%* | 22 | *13.8%* | 54 | *33.8%* | 52 | *32.5%* | 21 | *13.1%* |  |
| **Childhood social adversity, N, %** | |  |  |  |  |  |  |  |  |  |  |  |
|  | No adversities | 10 | *4.3%* | 29 | *12.6%* | 87 | *37.7%* | 75 | *32.5%* | 30 | *13.0%* | 0.467 |
|  | One or more adversities | 6 | *7.4%* | 14 | *17.3%* | 23 | *28.4%* | 27 | *33.3%* | 11 | *13.6%* |  |
| **Teenage pregnancy, N, %** | |  |  |  |  |  |  |  |  |  |  |  |
|  | No teenage pregnancy | 8 | *3.8%* | 26 | *12.5%* | 82 | *39.4%* | 67 | *32.2%* | 25 | *12.0%* | 0.167 |
|  | Teenage pregnancy (<20) | 8 | *7.7%* | 17 | *16.3%* | 28 | *26.9%* | 35 | *33.7%* | 16 | *15.4%* |  |
| **Parity, N, %** | |  |  |  |  |  |  |  |  |  |  |  |
|  | Nulliparous | 2 | *6.9%* | 3 | *10.3%* | 16 | *55.2%* | 5 | *17.2%* | 3 | *10.3%* | 0.364 |
|  | 1 to 3 births | 4 | *4.4%* | 10 | *11.1%* | 33 | *36.7%* | 33 | *36.7%* | 10 | *11.1%* |  |
|  | 4 or more births | 10 | *5.2%* | 30 | *15.5%* | 61 | *31.6%* | 64 | *33.2%* | 28 | *14.5%* |  |

P-values: chi2 for categorical/dichotomous covariates, anova for continuous covariates.

Table 1C: Population characteristics by age at natural menopause, region-specific – Albania

| Characteristics | | Age at natural menopause - categories | | | | | | | | | | P-value |
| --- | --- | --- | --- | --- | --- | --- | --- | --- | --- | --- | --- | --- |
|  |  | <40 (N=6) | | 40-44 (N=17) | | 45-49 (N=57) | | 50-54 (N=72) | | ≥55 (N=24) | |  |
| **Age, mean, SD** | | 68.67 | *3.67* | 68.76 | *2.8* | 68.81 | *3.19* | 69.39 | *2.98* | 69.00 | *3.26* | 0.834 |
| **Education, site specific tertiles, N, %** | |  |  |  |  |  |  |  |  |  |  |  |
|  | lowest tertile | 3 | *4.1%* | 13 | *17.6%* | 24 | *32.4%* | 25 | *33.8%* | 9 | *12.2%* | 0.070 |
|  | middle tertile | 1 | *1.6%* | 3 | *4.7%* | 24 | *37.5%* | 29 | *45.3%* | 7 | *10.9%* |  |
|  | highest tertile | 2 | *5.3%* | 1 | *2.6%* | 9 | *23.7%* | 18 | *47.4%* | 8 | *21.1%* |  |
| **Income, N, %** | |  |  |  |  |  |  |  |  |  |  |  |
|  | poor | 1 | *4.0%* | 3 | *12.0%* | 11 | *44.0%* | 9 | *36.0%* | 1 | *4.0%* | 0.405 |
|  | middle | 4 | *3.8%* | 9 | *8.6%* | 30 | *28.6%* | 42 | *40.0%* | 20 | *19.0%* |  |
|  | middle high/high | 1 | *2.2%* | 5 | *10.9%* | 16 | *34.8%* | 21 | *45.7%* | 3 | *6.5%* |  |
| **Height, mean, SD** | | 154.33 | *4.84* | 152.47 | *4.58* | 154.88 | *5.56* | 153.65 | *6.31* | 154.13 | *5.19* | 0.588 |
| **BMI, N, %** | |  |  |  |  |  |  |  |  |  |  |  |
|  | Normal or underweight (<25) | 1 | *4.2%* | 3 | *12.5%* | 9 | *37.5%* | 9 | *37.5%* | 2 | *8.3%* | 0.813 |
|  | Overweight (25.0-29.9) | 2 | *2.8%* | 6 | *8.3%* | 20 | *27.8%* | 30 | *41.7%* | 14 | *19.4%* |  |
|  | Obese (>=30) | 3 | *3.8%* | 8 | *10.0%* | 28 | *35.0%* | 33 | *41.3%* | 8 | *10.0%* |  |
| **Hysterectomy (post-menopausal), N, %** | |  |  |  |  |  |  |  |  |  |  |  |
|  | Had hysterectomy | 1 | *25.0%* | 0 | *0.0%* | 0 | *0.0%* | 1 | *25.0%* | 2 | *50.0%* | 0.021 |
|  | Did not have hysterectomy | 5 | *2.9%* | 17 | *9.9%* | 57 | *33.1%* | 71 | *41.3%* | 22 | *12.8%* |  |
| **HRT, ever, N, %** | |  |  |  |  |  |  |  |  |  |  |  |
|  | no | 6 | *3.6%* | 16 | *9.5%* | 55 | *32.7%* | 67 | *39.9%* | 24 | *14.3%* | 0.747 |
|  | yes | 0 | *0.0%* | 1 | *14.3%* | 2 | *28.6%* | 4 | *57.1%* | 0 | *0.0%* |  |
| **Smoking, last 15 years N, %** | |  |  |  |  |  |  |  |  |  |  |  |
|  | no | 3 | *2.0%* | 14 | *9.3%* | 52 | *34.4%* | 62 | *41.1%* | 20 | *13.2%* | 0.343 |
|  | yes | 2 | *8.3%* | 3 | *12.5%* | 5 | *20.8%* | 10 | *41.7%* | 4 | *16.7%* |  |
| **Childhood economic adversity, N, %** | |  |  |  |  |  |  |  |  |  |  |  |
|  | No adversities | 6 | *7.1%* | 7 | *8.3%* | 23 | *27.4%* | 36 | *42.9%* | 12 | *14.3%* | 0.081 |
|  | One or more adversities | 0 | *0.0%* | 10 | *10.9%* | 34 | *37.0%* | 36 | *39.1%* | 12 | *13.0%* |  |
| **Childhood social adversity, N, %** | |  |  |  |  |  |  |  |  |  |  |  |
|  | No adversities | 6 | *3.9%* | 14 | *9.1%* | 48 | *31.2%* | 65 | *42.2%* | 21 | *13.6%* | 0.673 |
|  | One or more adversities | 0 | *0.0%* | 3 | *13.6%* | 9 | *40.9%* | 7 | *31.8%* | 3 | *13.6%* |  |
| **Teenage pregnancy, N, %** | |  |  |  |  |  |  |  |  |  |  |  |
|  | No teenage pregnancy | 6 | *3.9%* | 15 | *9.7%* | 45 | *29.2%* | 66 | *42.9%* | 22 | *14.3%* | 0.185 |
|  | Teenage pregnancy (<20) | 0 | *0.0%* | 2 | *9.1%* | 12 | *54.5%* | 6 | *27.3%* | 2 | *9.1%* |  |
| **Parity, N, %** | |  |  |  |  |  |  |  |  |  |  |  |
|  | Nulliparous | 1 | *7.7%* | 0 | *0.0%* | 3 | *23.1%* | 6 | *46.2%* | 3 | *23.1%* | 0.064 |
|  | 1 to 3 births | 4 | *3.0%* | 10 | *7.6%* | 43 | *32.6%* | 60 | *45.5%* | 15 | *11.4%* |  |
|  | 4 or more births | 1 | *3.2%* | 7 | *22.6%* | 11 | *35.5%* | 6 | *19.4%* | 6 | *19.4%* |  |

* 1 missing value for HRT, 1 missing value for smoking

P-values: chi2 for categorical/dichotomous covariates, anova for continuous covariates.

Table 2A: Distribution (or mean) of exposure variable and population characteristics by gait speed, region-specific

|  |  | **Canada (N=267)** | | | **Latin America (N=305)** | | | **Albania (N=169)** | | |
| --- | --- | --- | --- | --- | --- | --- | --- | --- | --- | --- |
| Characteristics | | Mean | SD | P-Value | Mean | SD | P-Value | Mean | SD | P-Value |
| **Age at natural menopause** | |  |  | **0.46** |  |  | **0.48** |  |  | **0.20** |
|  | <40 | 0.94 | 0.18 | 0.103 | 0.73 | 0.18 | 0.501 | 0.81 | 0.1 | 0.882 |
|  | 40-44 | 1.06 | 0.18 | 0.776 | 0.79 | 0.2 | 0.382 | 0.88 | 0.18 | 0.223 |
|  | 45-49 | 1.08 | 0.25 | 0.861 | 0.8 | 0.21 | 0.229 | 0.84 | 0.29 | 0.333 |
|  | 50-54 | 1.07 | 0.22 | ref | 0.76 | 0.17 | ref | 0.79 | 0.27 | ref |
|  | ≥55 | 1.09 | 0.23 | 0.652 | 0.81 | 0.2 | 0.25 | 0.94 | 0.31 | 0.021 |
| **Study site** | |  |  |  |  |  |  |  |  |  |
|  | Kingston | 1.09 | 0.23 | ref |  |  |  |  |  |  |
|  | St Hyacinthe | 1.05 | 0.22 | 0.078 |  |  |  |  |  |  |
|  | Tirana |  |  |  |  |  |  | 0.84 | 0.27 | - |
|  | Manizales |  |  |  | 0.82 | 0.17 | ref |  |  |  |
|  | Natal |  |  |  | 0.74 | 0.21 | <0.001 |  |  |  |
| **Age** (each additional year) | | -0.02 |  | <0.001 | -0.01 |  | 0.014 | -0.02 |  | 0.017 |
| **Education, site specific tertiles** | |  |  | **0.006** |  |  | **0.006** |  |  | **0.002** |
|  | lowest tertile | 1.02 | 0.22 | <0.001 | 0.75 | 0.18 | 0.010 | 0.77 | 0.28 | <0.001 |
|  | middle tertile | 1.10 | 0.25 | 0.073 | 0.79 | 0.20 | 0.254 | 0.83 | 0.22 | 0.014 |
|  | highest tertile | 1.15 | 0.19 | ref | 0.82 | 0.18 | ref | 0.97 | 0.30 | ref |
|  | |  |  |  |  |  |  |  |  |  |
| **Income*** | |  |  | **<0.001** |  |  | **0.04** |  |  | **0.07** |
|  | poor | 1.00 | 0.23 | <0.001 | 0.81 | 0.17 | 0.912 | 0.75 | 0.32 | 0.027 |
|  | middle | 1.09 | 0.22 | 0.073 | 0.75 | 0.20 | 0.096 | 0.83 | 0.28 | 0.013 |
|  | high | 1.15 | 0.19 | ref | 0.81 | 0.21 | ref | 0.9 | 0.22 | ref |
| **Height (each cm increase)** | | 0.01 |  | 0.005 | 0.01 |  | 0.001 | 0.004 |  | 0.247 |
|  |  |  |  |  |  |  |  |  |  |  |
| **BMI** |  |  |  | **0.001** |  |  | **<0.001** |  |  | **0.42** |
|  | Normal or under weight (<25) | 1.13 | 0.22 | ref | 0.83 | 0.21 | ref | 0.81 | 0.22 | ref |
|  | Overweight (25.0-29.9) | 1.07 | 0.19 | 0.106 | 0.79 | 0.17 | 0.090 | 0.87 | 0.25 | 0.354 |
|  | Obese (>=30) | 1.00 | 0.26 | <0.001 | 0.73 | 0.20 | <0.001 | 0.81 | 0.31 | 0.924 |
| **Hysterectomy (post-menopausal)** | |  |  |  |  |  |  |  |  |  |
|  | Had hysterectomy | 1.01 | 0.29 | 0.108 | 0.79 | 0.19 | 0.892 | 0.88 | 0.37 | 0.799 |
|  | Did not have hysterectomy | 1.08 | 0.22 | ref | 0.78 | 0.19 | ref | 0.83 | 0.27 | ref |
| **HRT, ever** | |  |  |  |  |  |  |  |  |  |
|  | never (n=113) | 1.06 | 0.23 | 0.588 | 0.77 | 0.19 | 0.043 | 0.84 | 0.28 | 0.343 |
|  | ever (N=172) | 1.08 | 0.23 | ref | 0.83 | 0.17 | ref | 0.74 | 0.06 | ref |
| **Smoking, currently** | |  |  |  |  |  |  |  |  |  |
|  | no (n=267) | 1.08 | 0.22 | ref | 0.78 | 0.19 | ref | 0.82 | 0.27 | ref |
|  | yes (n=18) | 0.95 | 0.22 | 0.017 | 0.78 | 0.19 | 0.972 | 0.94 | 0.29 | 0.063 |
| **Childhood economic adversity** | |  |  |  |  |  |  |  |  |  |
|  | No adversities | 1.09 | 0.22 | ref | 0.81 | 0.19 | ref | 0.90 | 0.29 | ref |
|  | One or more adversities | 1.05 | 0.24 | 0.186 | 0.76 | 0.19 | 0.043 | 0.78 | 0.25 | 0.004 |
| **Childhood social adversity** | |  |  |  |  |  |  |  |  |  |
|  | No adversities | 1.10 | 0.22 | ref | 0.79 | 0.19 | ref | 0.85 | 0.28 | ref |
|  | One or more adversities | 1.01 | 0.24 | 0.002 | 0.76 | 0.21 | 0.213 | 0.7 | 0.21 | 0.017 |
| **Teenage pregnancy** | |  |  |  |  |  |  |  |  |  |
|  | No teenage pregnancy | 1.08 | 0.22 | ref | 0.79 | 0.18 | ref | 0.85 | 0.28 | ref |
|  | Teenage pregnancy (<20) | 1.02 | 0.29 | 0.227 | 0.77 | 0.21 | 0.405 | 0.72 | 0.24 | 0.050 |
|  | |  |  |  |  |  |  |  |  |  |
| **Parity** | |  |  | **0.67** |  |  | **0.80** |  |  | **0.38** |
|  | Nulliparous | 1.07 | 0.24 | 0.889 | 0.80 | 0.16 | 0.440 | 0.86 | 0.16 | 0.919 |
|  | 1 to 3 births | 1.08 | 0.22 | ref | 0.77 | 0.19 | ref | 0.85 | 0.28 | ref |
|  | 4 or more births | 1.05 | 0.22 | 0.430 | 0.78 | 0.20 | 0.647 | 0.77 | 0.27 | 0.174 |

Table 2B: Distribution (or mean) of exposure variable and population characteristics by grip strength, region-specific

|  |  | **Canada (N=267)** | | | **Latin America (N=305)** | | | **Albania (N=169)** | | |
| --- | --- | --- | --- | --- | --- | --- | --- | --- | --- | --- |
| Characteristics | | Mean | SD | P-Value | Mean | SD | P-Value | Mean | SD | P-Value |
| **Age at natural menopause** | |  |  | **0.20** |  |  | **0.11** |  |  | **0.21** |
|  | <40 | 20.89 | 3.14 | 0.075 | 17.72 | 4.41 | 0.048 | 16.34 | 7.35 | 0.11 |
|  | 40-44 | 24.35 | 8.06 | 0.964 | 18.64 | 4.41 | 0.074 | 20.71 | 4.71 | 0.78 |
|  | 45-49 | 22.74 | 5.36 | 0.073 | 19.72 | 4.72 | 0.548 | 21.09 | 6.03 | 0.406 |
|  | 50-54 | 24.29 | 5.02 | ref | 20.09 | 4.25 | ref | 20.3 | 4.63 | ref |
|  | ≥55 | 23.3 | 5.6 | 0.254 | 20.44 | 4.09 | 0.673 | 22.22 | 5.35 | 0.137 |
| **Study site** | |  |  |  |  |  |  |  |  |  |
|  | Kingston | 23.07 | *5.75* | *ref* |  |  |  |  |  |  |
|  | St Hyacinthe | 24.22 | *5.12* | 0.092 |  |  |  |  |  |  |
|  | Tirana |  |  |  |  |  |  | 20.75 | 5.35 | - |
|  | Manizales |  |  |  | 20.31 | 4.15 | *ref* |  |  |  |
|  | Natal |  |  |  | 19.07 | 4.68 | 0.015 |  |  |  |
| **Age (**each additional year**)** | | -0.35 |  | 0.006 | -0.14 |  | 0.130 | -0.39 |  | 0.004 |
|  | |  |  |  |  |  |  |  |  |  |
| **Education, site specific tertiles** | |  |  | **0.78** |  |  | **0.86** |  |  | **0.31** |
|  | lowest tertile | 23.27 | *6.06* | 0.533 | 19.69 | *4.69* | 0.753 | 20.79 | *5.45* | 0.370 |
|  | middle tertile | 23.76 | *5.44* | 0.949 | 19.53 | *4.48* | 0.589 | 20.06 | *5.50* | 0.132 |
|  | highest tertile | 23.82 | *4.57* | *ref* | 19.90 | *4.03* | ref | 21.74 | *4.86* | *ref* |
|  | |  |  |  |  |  |  |  |  |  |
| **Income*** | |  |  | **0.26** |  |  | **0.19** |  |  | **0.06** |
|  | poor | 22.85 | *4.79* | 0.320 | 20.18 | *4.23* | 0.752 | 18.76 | *5.64* | 0.020 |
|  | middle | 24.15 | *6.13* | 0.607 | 19.21 | *4.58* | 0.362 | 20.75 | *4.96* | 0.239 |
|  | high | 23.71 | *5.38* | *ref* | 19.92 | *4.61* | ref | 21.89 | *5.84* | *ref* |
| **Height (each cm increase)** | | 0.19 |  | 0.001 | 0.23 |  | <0.001 | 0.17 |  | 0.018 |
| **BMI** |  |  |  | **0.61** |  |  | **0.56** |  |  | **0.92** |
|  | Normal or underweight (<25) | 23.86 | *5.56* | *ref* | 19.53 | *3.98* | *ref* | 20.78 | *4.43* | *ref* |
|  | Overweight (25.0-29.9) | 23.73 | *5.69* | 0.875 | 19.97 | *4.64* | 0.467 | 20.56 | *6.05* | 0.862 |
|  | Obese (>=30) | 23.01 | *5.20* | 0.330 | 19.35 | *4.65* | 0.795 | 20.90 | *4.99* | 0.924 |
| **Hysterectomy (post-menopausal)** | |  |  |  |  |  |  |  |  |  |
|  | Did not have hysterectomy | 22.67 | *5.12* | *ref* | 21.03 | *4.54* | *ref* | 21.00 | *1.41* | *ref* |
|  | Had hysterectomy | 23.71 | *5.55* | 0.311 | 19.48 | *4.42* | 0.046 | 20.74 | *5.38* | 0.947 |
| **HRT, ever** | |  |  |  |  |  |  |  |  |  |
|  | never | 22.78 | *5.46* | 0.054 | 19.43 | *4.35* | 0.028 | 20.8 | *5.40* | 0.507 |
|  | ever | 24.11 | *5.49* | *ref* | 20.96 | *4.84* | ref | 19.43 | *3.87* | *ref* |
| **Smoking, currently** | |  |  |  |  |  |  |  |  |  |
|  | no | 23.78 | *5.36* | *ref* | 19.71 | *4.42* | *ref* | 20.59 | *5.21* | *ref* |
|  | yes | 20.77 | *6.73* | 0.024 | 19.34 | *4.90* | 0.676 | 21.85 | *6.20* | 0.302 |
| **Childhood economic adversity** | |  |  |  |  |  |  |  |  |  |
|  | No adversities | 23.70 | *5.44* | *ref* | 19.55 | *4.39* | *ref* | 22.04 | *4.95* | *ref* |
|  | One or more adversities | 23.33 | *5.66* | 0.613 | 19.79 | *4.53* | 0.647 | 19.58 | *5.45* | 0.002 |
| **Childhood social adversity** | |  |  |  |  |  |  |  |  |  |
|  | No adversities | 23.61 | *5.42* | *ref* | 19.87 | *4.43* | *ref* | 21.13 | *4.97* | *ref* |
|  | One or more adversities | 23.5 | *5.73* | 0.884 | 19.13 | *4.54* | 0.204 | 18.18 | *7.06* | 0.015 |
| **Teenage pregnancy** | |  |  |  |  |  |  |  |  |  |
|  | No teenage pregnancy | 23.71 | *5.43* | *ref* | 19.41 | *4.6* | *ref* | 20.88 | *5.33* | *ref* |
|  | Teenage pregnancy (<20) | 22.09 | *6.25* | 0.196 | 20.2 | *4.14* | 0.149 | 19.86 | *5.51* | 0.408 |
| **Parity** | |  |  | **0.37** |  |  | **0.25** |  |  | **0.85** |
|  | Nulliparous | 23.93 | *6.43* | 0.825 | 19.07 | *4.52* | 0.920 | 21.25 | *5.59* | 0.697 |
|  | 1 to 3 births | 23.72 | *5.46* | *ref* | 19.17 | *4.48* | *ref* | 20.62 | *5.36* | *ref* |
|  | 4 or more births | 22.41 | *4.41* | 0.192 | 20 | *4.43* | 0.147 | 21.1 | *5.35* | 0.656 |

Table 3A: Multivariate linear regression models presenting the association of ANM and gait speed, region-specific

|  |  | **Canada** | | | | | | **Latin America** | | | | | | **Albania** | | | | | |
| --- | --- | --- | --- | --- | --- | --- | --- | --- | --- | --- | --- | --- | --- | --- | --- | --- | --- | --- | --- |
|  |  | **Adjusted for age and site** | | | **Adjusted model (parsimonious)** | | | **Adjusted for age and site** | | | **Adjusted model (parsimonious)** | | | **Adjusted for age** | | | **Adjusted model (parsimonious)** | | |
|  |  | **(N=267)** | | | **(N=267)** | | | **(N=305)** | | | **(N=305)** | | | **(N=169)** | | | **(N=169)** | | |
|  |  | **B** | **95%CI** | | **B** | **95%CI** | | **B** | **95%CI** | | **B** | **95%CI** | | **B** | **95%CI** | | **B** | **95%CI** | |
| **Age at natural menopause** | |  |  |  |  |  |  |  |  |  |  |  |  |  |  |  |  |  |  |
|  | <40 | -0.12 | -0.28 | 0.03 | -0.06 | -0.21 | 0.09 | -0.03 | -0.13 | 0.07 | -0.03 | -0.13 | 0.07 | 0.01 | -0.23 | 0.26 | -0.06 | -0.30 | 0.18 |
|  | 40-44 | -0.03 | -0.13 | 0.08 | 0.01 | -0.08 | 0.11 | 0.04 | -0.02 | 0.11 | 0.04 | -0.02 | 0.11 | 0.08 | -0.06 | 0.23 | 0.13 | -0.01 | 0.28 |
|  | 45-49 | 0.02 | -0.05 | 0.09 | 0.04 | -0.03 | 0.10 | 0.04 | -0.01 | 0.09 | 0.04 | 0.00 | 0.09 | 0.04 | -0.06 | 0.13 | 0.07 | -0.03 | 0.16 |
|  | 50-54 | *ref* |  |  | *ref* |  |  | *ref* |  |  | *ref* |  |  | *ref* |  |  | *ref* |  |  |
|  | ≥55 | 0.03 | -0.03 | 0.10 | 0.03 | -0.03 | 0.10 | 0.04 | -0.03 | 0.11 | 0.04 | -0.03 | 0.11 | 0.15 | 0.02 | 0.28 | 0.13 | 0.01 | 0.25 |
|  |  |  |  | ***0.29*** |  |  | ***0.49*** |  |  | ***0.34*** |  |  | ***0.25*** |  |  | ***0.23*** |  |  | ***0.10*** |
| Age (years) | | -0.02 | -0.03 | -0.01 | -0.02 | -0.03 | -0.01 | -0.01 | -0.02 | 0.00 | -0.01 | -0.02 | 0.00 | -0.02 | -0.03 | 0.00 | -0.01 | -0.03 | 0.00 |
| Study site | |  |  |  |  |  |  |  |  |  |  |  |  |  |  |  |  |  |  |
|  | Kingston | *ref* |  |  | *ref* |  |  |  |  |  |  |  |  |  |  |  |  |  |  |
|  | St Hyacinthe | -0.07 | -0.12 | -0.01 | -0.02 | -0.08 | 0.03 |  |  |  |  |  |  |  |  |  |  |  |  |
|  | Tirana |  |  |  |  |  |  |  |  |  |  |  |  |  |  |  |  |  |  |
|  | Manizales |  |  |  |  |  |  | *ref* |  |  | *ref* |  |  |  |  |  |  |  |  |
|  | Natal |  |  |  |  |  |  | -0.08 | -0.13 | -0.04 | -0.08 | -0.13 | -0.04 |  |  |  |  |  |  |
| Education, site specific tertiles | |  |  |  |  |  |  |  |  |  |  |  |  |  |  |  |  |  |  |
|  | lowest tertile |  |  |  |  |  |  |  |  |  |  |  |  |  |  |  | -0.19 | -0.29 | -0.08 |
|  | middle tertile |  |  |  |  |  |  |  |  |  |  |  |  |  |  |  | -0.12 | -0.23 | -0.02 |
|  | highest tertile |  |  |  |  |  |  |  |  |  |  |  |  |  |  |  | *ref* |  |  |
| Income, site specific | |  |  |  |  |  |  |  |  |  |  |  |  |  |  |  |  |  |  |
|  | Low |  |  |  | -0.09 | -0.16 | -0.01 |  |  |  |  |  |  |  |  |  |  |  |  |
|  | Middle |  |  |  | -0.03 | -0.10 | 0.03 |  |  |  |  |  |  |  |  |  |  |  |  |
|  | High |  |  |  | *ref* |  |  |  |  |  |  |  |  |  |  |  |  |  |  |
| Height (cm) | |  |  |  |  |  |  |  |  |  | 0.01 | 0.00 | 0.01 |  |  |  |  |  |  |
| BMI | |  |  |  |  |  |  |  |  |  |  |  |  |  |  |  |  |  |  |
|  | Normal weight or underweight |  |  |  | *ref* |  |  |  |  |  | *ref* |  |  |  |  |  |  |  |  |
|  | Overweight |  |  |  | -0.06 | -0.11 | 0.00 |  |  |  | -0.03 | -0.08 | 0.02 |  |  |  |  |  |  |
|  | Obese |  |  |  | -0.12 | -0.18 | -0.05 |  |  |  | -0.07 | -0.13 | -0.02 |  |  |  |  |  |  |
| Post-menopausal hysterectomy (ref: no) | |  |  |  |  |  |  |  |  |  |  |  |  |  |  |  |  |  |  |
| Ever used HRT (ref: yes, ever) | |  |  |  |  |  |  |  |  |  |  |  |  |  |  |  |  |  |  |
| Currently smoking (ref: no) | |  |  |  | -0.13 | -0.23 | -0.03 |  |  |  |  |  |  |  |  |  | 0.11 | -0.01 | 0.22 |
| Childhood economic adversity (ref: none) | |  |  |  |  |  |  |  |  |  |  |  |  |  |  |  | -0.09 | -0.17 | -0.01 |
| Childhood social adversity (ref: none) | |  |  |  | -0.06 | -0.12 | -0.01 |  |  |  |  |  |  |  |  |  |  |  |  |
| Adolescent pregnancy (ref: no) | |  |  |  |  |  |  |  |  |  |  |  |  |  |  |  |  |  |  |
| Parity | |  |  |  |  |  |  |  |  |  |  |  |  |  |  |  |  |  |  |
|  | Nulliparous |  |  |  |  |  |  |  |  |  |  |  |  |  |  |  |  |  |  |
|  | 1-3 births |  |  |  |  |  |  |  |  |  |  |  |  |  |  |  |  |  |  |
|  | 4 or more births |  |  |  |  |  |  |  |  |  |  |  |  |  |  |  |  |  |  |
|  |  |  |  |  |  |  |  |  |  |  |  |  |  |  |  |  |  |  |  |
| R-squared | | 0.10 |  |  | 0.20 |  |  | 0.08 |  |  | 0.15 |  |  | 0.07 |  |  | 0.19 |  |  |
| Adj R-squared | | 0.07 |  |  | 0.17 |  |  | 0.06 |  |  | 0.12 |  |  | 0.04 |  |  | 0.14 |  |  |
| Constant | | 2.70 |  |  | 2.70 |  |  | 1.51 |  |  | 0.45 |  |  | 1.89 |  |  | 1.91 |  |  |

Table 4A: Multivariate linear regression models presenting the association of ANM and grip strength, region specific analysis

|  |  | **Canada** | | | | | | **Latin America** | | | | | | **Albania** | | | | | |
| --- | --- | --- | --- | --- | --- | --- | --- | --- | --- | --- | --- | --- | --- | --- | --- | --- | --- | --- | --- |
|  |  | **Adjusted for age and site** | | | **Adjusted model (parsimonious)** | | | **Adjusted for age and site** | | | **Adjusted model (parsimonious)** | | | **Adjusted for age** | | | **Adjusted model (parsimonious)** | | |
|  |  | **(N=267)** | | | **(N=267)** | | | **(N=305)** | | | **(N=305)** | | | **(N=171)** | | | **(N=171)** | | |
|  |  | **B** | **95%CI** | | **B** | **95%CI** | | **B** | **95%CI** | | **B** | **95%CI** | | **B** | **95%CI** | | **B** | **95%CI** | |
| **Age at natural menopause** | |  |  |  |  |  |  |  |  |  |  |  |  |  |  |  |  |  |  |
|  | <40 | -2.88 | -6.60 | 0.84 | -2.74 | -6.33 | 0.84 | -2.34 | -4.68 | -0.01 | -2.45 | -4.66 | -0.25 | -4.11 | -8.87 | 0.65 | -5.55 | -10.11 | -0.99 |
|  | 40-44 | -0.06 | -2.60 | 2.48 | 0.28 | -2.19 | 2.75 | -1.30 | -2.89 | 0.29 | -1.11 | -2.61 | 0.38 | 0.17 | -2.62 | 2.95 | 0.79 | -1.86 | 3.43 |
|  | 45-49 | -1.50 | -3.18 | 0.18 | -1.21 | -2.84 | 0.41 | -0.30 | -1.51 | 0.90 | 0.02 | -1.13 | 1.16 | 0.57 | -1.27 | 2.42 | 0.81 | -0.94 | 2.57 |
|  | 50-54 | *ref* |  |  | *ref* |  |  | *ref* |  |  | *ref* |  |  | *ref* |  |  | *ref* |  |  |
|  | ≥55 | -0.82 | -2.52 | 0.89 | -1.10 | -2.74 | 0.55 | 0.34 | -1.29 | 1.98 | 0.47 | -1.07 | 2.00 | 1.78 | -0.69 | 4.26 | 1.89 | -0.45 | 4.23 |
|  | ***P value*** |  |  | ***0.30*** |  |  | ***0.32*** |  |  | **0.15** |  |  | **0.08** |  |  | **0.21** |  |  | **0.04** |
| Age (years) | | -0.29 | -0.55 | -0.04 | -0.27 | -0.52 | -0.02 | -0.11 | -0.29 | 0.07 | -0.10 | -0.28 | 0.07 | -0.38 | -0.64 | -0.12 | -0.37 | -0.61 | -0.12 |
| Study site | |  |  |  |  |  |  |  |  |  |  |  |  |  |  |  |  |  |  |
|  | Kingston | *ref* |  |  | *ref* |  |  |  |  |  |  |  |  |  |  |  |  |  |  |
|  | St Hyacinthe | 1.02 | -0.31 | 2.35 | 2.05 | 0.70 | 3.40 |  |  |  |  |  |  |  |  |  |  |  |  |
|  | Tirana |  |  |  |  |  |  |  |  |  |  |  |  |  |  |  |  |  |  |
|  | Manizales |  |  |  |  |  |  | *ref* |  |  | *ref* |  |  |  |  |  |  |  |  |
|  | Natal |  |  |  |  |  |  | -1.25 | -2.24 | -0.26 | -1.52 | -2.47 | -0.57 |  |  |  |  |  |  |
| Education, site specific tertiles | |  |  |  |  |  |  |  |  |  |  |  |  |  |  |  |  |  |  |
|  | lowest tertile |  |  |  |  |  |  |  |  |  |  |  |  |  |  |  |  |  |  |
|  | middle tertile |  |  |  |  |  |  |  |  |  |  |  |  |  |  |  |  |  |  |
|  | highest tertile |  |  |  |  |  |  |  |  |  |  |  |  |  |  |  |  |  |  |
| Income, site specific | |  |  |  |  |  |  |  |  |  |  |  |  |  |  |  |  |  |  |
|  | Low |  |  |  |  |  |  |  |  |  |  |  |  |  |  |  |  |  |  |
|  | Middle |  |  |  |  |  |  |  |  |  |  |  |  |  |  |  |  |  |  |
|  | High |  |  |  |  |  |  |  |  |  |  |  |  |  |  |  |  |  |  |
| Height (cm) | |  |  |  | 0.22 | 0.11 | 0.34 |  |  |  | 0.25 | 0.16 | 0.33 |  |  |  | 0.14 | 0.01 | 0.28 |
| BMI | |  |  |  |  |  |  |  |  |  |  |  |  |  |  |  |  |  |  |
|  | Normal weight or underweight |  |  |  |  |  |  |  |  |  |  |  |  |  |  |  |  |  |  |
|  | Overweight |  |  |  |  |  |  |  |  |  |  |  |  |  |  |  |  |  |  |
|  | Obese |  |  |  |  |  |  |  |  |  |  |  |  |  |  |  |  |  |  |
| Post-menopausal hysterectomy (ref: no) | |  |  |  |  |  |  |  |  |  | 1.58 | 0.15 | 3.00 |  |  |  |  |  |  |
| Ever used HRT (ref: yes, ever) | |  |  |  |  |  |  |  |  |  | -1.16 | -2.45 | 0.14 |  |  |  |  |  |  |
| Currently smoking (ref: no) | |  |  |  | -3.19 | -5.76 | -0.61 |  |  |  |  |  |  |  |  |  |  |  |  |
| Childhood economic adversity (ref: none) | |  |  |  |  |  |  |  |  |  |  |  |  |  |  |  | -2.23 | -3.80 | -0.67 |
| Childhood social adversity (ref: none) | |  |  |  |  |  |  |  |  |  |  |  |  |  |  |  | -2.62 | -4.91 | -0.34 |
| Adolescent pregnancy (ref: no) | |  |  |  |  |  |  |  |  |  | 0.99 | -0.01 | 1.99 |  |  |  |  |  |  |
| Parity | |  |  |  |  |  |  |  |  |  |  |  |  |  |  |  |  |  |  |
|  | Nulliparous |  |  |  |  |  |  |  |  |  |  |  |  |  |  |  |  |  |  |
|  | 1-3 births |  |  |  |  |  |  |  |  |  |  |  |  |  |  |  |  |  |  |
|  | 4 or more births |  |  |  |  |  |  |  |  |  |  |  |  |  |  |  |  |  |  |
|  |  |  |  |  |  |  |  |  |  |  |  |  |  |  |  |  |  |  |  |
| R-squared | | 0.05 |  |  | 0.13 |  |  | 0.05 |  |  | 0.17 |  |  | 0.08 |  |  | 0.19 |  |  |
| Adj R-squared | | 0.03 |  |  | 0.10 |  |  | 0.03 |  |  | 0.15 |  |  | 0.05 |  |  | 0.15 |  |  |
| Constant | | 44.08 |  |  | 6.44 |  |  | 28.29 |  |  | -8.59 |  |  | 46.64 |  |  | 24.93 |  |  |
